# Left Inferior Pulmonary Vein Thrombosis and Secondary Lung Infarction Following Radiofrequency Catheter Ablation: A Diagnostic Challenge

**DOI:** 10.7759/cureus.116857

**Published:** 2026-09-24

**Authors:** Karen M'bie, Pascale Bohy, Aurore Margot

**Affiliations:** 1 Radiology, Université Libre de Bruxelles, Bruxelles, BEL; 2 Radiology, Erasmus Hospital, Bruxelles, BEL

**Keywords:** atrial fibrillation, computed tomography pulmonary angiography, hemoptysis, lung infarction, pulmonary vein thrombosis, radiofrequency catheter ablation

## Abstract

Radiofrequency catheter ablation of atrial fibrillation is a common procedure, but it can be complicated by pulmonary vein thrombosis (PVT), a rare and underdiagnosed entity. We report the case of a 39-year-old female presenting with hemoptysis on day four post-ablation. The initial chest computed tomography angiography was interpreted as isolated alveolar hemorrhage, but the follow-up examination at day 31 revealed complete left inferior PVT associated with a secondary pulmonary infarction. Given the non-specific clinical presentation of post-ablation respiratory symptoms, systematic radiological evaluation of the pulmonary veins with adequate vascular contrast enhancement is essential to rule out PVT, a rare yet potentially grave complication, and prevent catastrophic systemic thromboembolic events or irreversible parenchymal damage.

## Introduction

Pulmonary vein isolation (PVI) via catheter ablation, particularly radiofrequency catheter ablation (RFCA), has become the cornerstone and reference treatment for patients with symptomatic atrial fibrillation (AF) refractory to medical therapy [1]. The procedure aims to eliminate ectopic arrhythmogenic triggers, abnormal electrical impulses originating near the pulmonary vein junctions, by delivering thermal energy to the venous entry sites into the left atrium, known as the ostia and antrum [1,2]. Although RFCA is highly effective, major complications occur in 2.9% to 6.3% of procedures [1]. Direct thermal application to the venous wall carries an inherent risk of vascular injury; heat delivery damages the delicate endothelial lining, inducing a localized inflammatory wall reaction, disrupting local antithrombotic properties, and potentially activating the intraluminal coagulation cascade [2].

Pulmonary vein stenosis (PVS) is the most frequently described vascular complication [2], whereas true pulmonary vein thrombosis (PVT) is considerably rarer and frequently underdiagnosed [3]. Both clinical entities share a delayed onset, typically manifesting from a few weeks to several months after the intervention [4].

PVS is characterized by a progressive, subacute, or chronic fibrotic narrowing of the venous lumen. Its prevalence varied widely in historical series, ranging from 3% to 42%, depending on the ablation technique, measurement methodology, and follow-up interval [2]. Fortunately, thanks to technological advances, this incidence has decreased significantly to less than 1% (approaching 0%) [5,6]. This dramatic reduction is a direct result of utilizing three-dimensional (3D) electroanatomical mapping systems and performing wide antral circumferential ablation [1,5]. These techniques allow operators to deliver thermal energy far from the PV ostia, avoiding direct ablation inside the vascular lumen [1,5]. Nevertheless, despite these safer contemporary practices, the risk of venous wall injury persists [1].

In contrast to PVS, PVT is defined by the acute or subacute formation of an intraluminal thrombus within one or more pulmonary veins [3]. Its pathophysiology following RFCA involves direct thermal injury to the venous endothelium, resulting in the loss of local antithrombotic properties, a severe inflammatory vascular wall reaction, and subsequent activation of the intraluminal coagulation cascade [7]. The clinical manifestations of PVT are highly non-specific, including dyspnea, chest pain, cough, and hemoptysis, and frequently mimic much more common respiratory conditions, such as infectious pneumonia, arterial pulmonary embolism, or lung malignancy, thereby leading to significant diagnostic delays and erroneous therapeutic paths [2,7,8]. Computed tomography pulmonary angiography (CTPA) is the imaging modality of choice: it enables direct visualization of the intraluminal thrombus as a filling defect, demonstrates downstream secondary pulmonary venous infarction, and rules out concomitant arterial thromboembolism [3,9]. Although initial management relies on optimized systemic anticoagulation [9], the treatment of these severe obstructions may sometimes require invasive revascularization via percutaneous balloon angioplasty and stenting [10].

Here, we report the case of a 39-year-old female who developed complete thrombosis of the left inferior pulmonary vein (LIPV) complicated by secondary pulmonary infarction, presenting with hemoptysis and persistent chest pain after undergoing RFCA for AF. This classic yet rare complication highlights the diagnostic pitfalls in acute care settings, particularly when early post-procedural respiratory symptoms mimic more common pulmonary conditions, leading to potential misinterpretation by on-call clinicians and young radiologists.

## Case presentation

A 39-year-old female with a history of paroxysmal AF underwent electrical PVI via RFCA.

Four days postoperatively, the patient presented with chest pain and hemoptysis, prompting an emergency chest computed tomography (CT) scan. The imaging demonstrated a posterior consolidation in the left lower lobe (LLL) associated with surrounding ground-glass opacities extending minimally to the posterior portion of the left upper lobe (LUL) (Figure 1A-1C). There was no evidence of arterial pulmonary embolism, active contrast extravasation, or vascular fistula (Figure 1D). Given the immediate postoperative setting, a localized alveolar hemorrhage of mechanical or traumatic origin was considered the primary diagnostic hypothesis. The initial management consisted of close in-hospital clinical monitoring and immediate suspension of her prophylactic anticoagulant therapy, apixaban (5 mg twice daily). Concurrently, empirical antibiotic therapy with amoxicillin/clavulanic acid was administered for one week to cover potential pneumonia and prevent secondary infection of the consolidated lung parenchyma.

**Figure 1:**
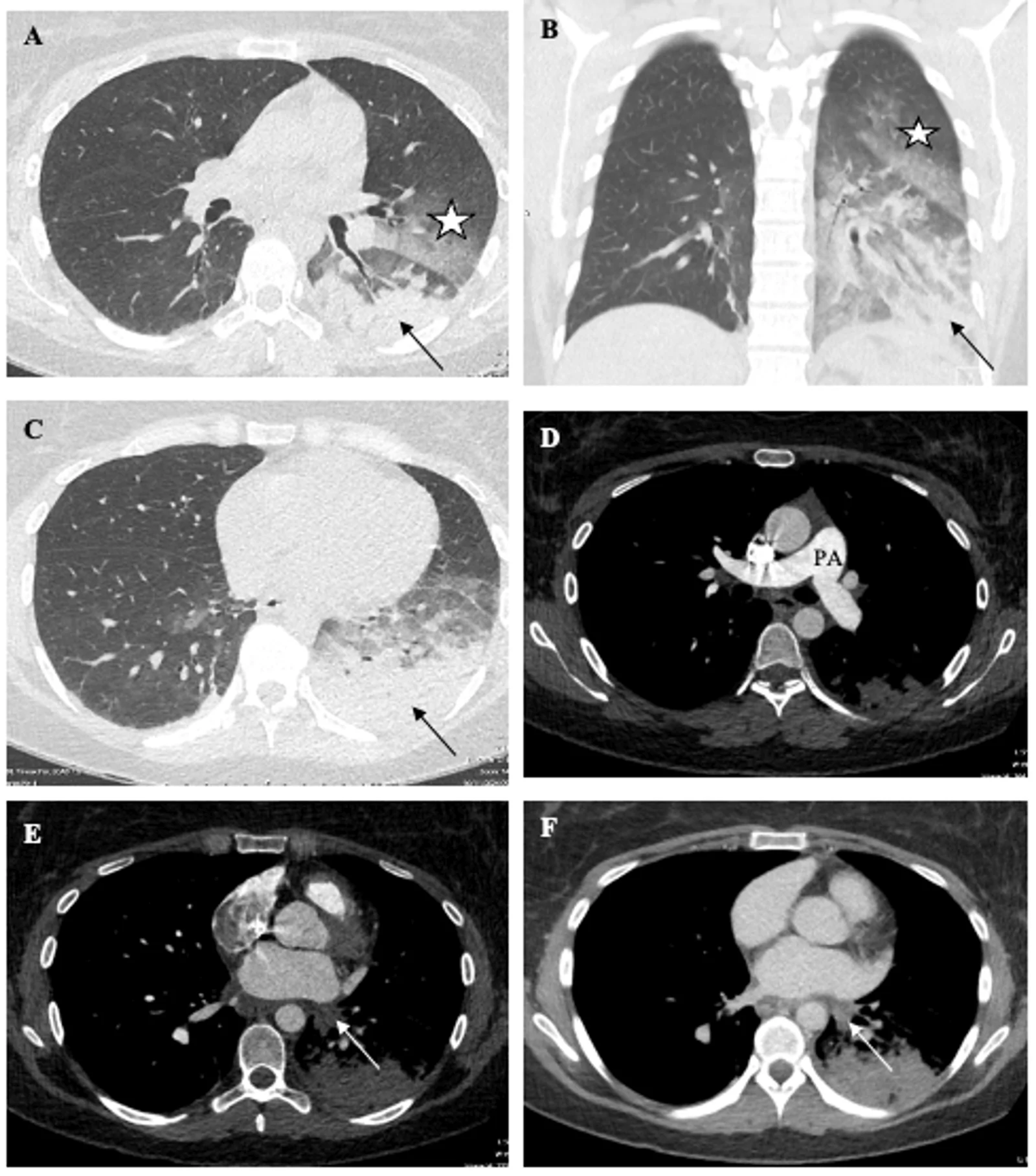
Initial chest computed tomography angiography (CTPA) performed emergently four days post-ablation. Panels A, B, and C (axial lung window): Large area of alveolar consolidation (black arrows) in the posterior segment of the left lower lobe (LLL), surrounded by adjacent ground-glass opacities (white stars with black outlines) extending minimally to the posterior segment of the left upper lobe (LUL). These findings are highly characteristic of localized alveolar hemorrhage secondary to early venous pulmonary infarction. Panel D (axial mediastinal window, arterial phase): Patency of the main and lobar pulmonary arteries (pulmonary artery, PA), ruling out a concomitant arterial pulmonary embolism. No active contrast extravasation or vascular fistula is visualized. Panel E (axial mediastinal window, arterial phase) and Panel F (axial mediastinal window, delayed venous phase, retrospective review): A retrospective analysis reveals an intraluminal filling defect (hypodensity) (white arrows) at the ostium of the left inferior pulmonary vein (LIPV), demonstrating that the thrombosis was already established at day 4 post-procedure.

Following the complete resolution of the hemoptysis, therapeutic anticoagulation with enoxaparin (60 mg twice daily) was safely initiated on day 11 post-ablation for a planned duration of one month, allowing for the patient's discharge.

At day 31, due to persistent chest pain, a follow-up chest computed tomography angiography (CTPA) was requested by the managing physician to rule out a sequelar vascular malformation or fistula. The CTPA revealed a distinct intraluminal filling defect (complete lack of enhancement) in the LIPV, confirming a complete PVT (Figure 2E). Concurrently, the lung parenchyma demonstrated a peripheral wedge-shaped consolidation in the LLL, secondary to a pulmonary venous infarction, which showed partial regression compared to the initial infiltrates observed on day 4 (Figure 2A-2C). No associated arterial pulmonary embolism was found (Figure 2D). Retrospective evaluation of our patient's day 4 scan confirmed that an early filling defect was already visible at the ostium of the LIPV on both the chronic thromboembolic pulmonary hypertension (CTEPH) arterial phase (Figure 1E) and the 90-second delayed venous phase (Figure 1F).

**Figure 2:**
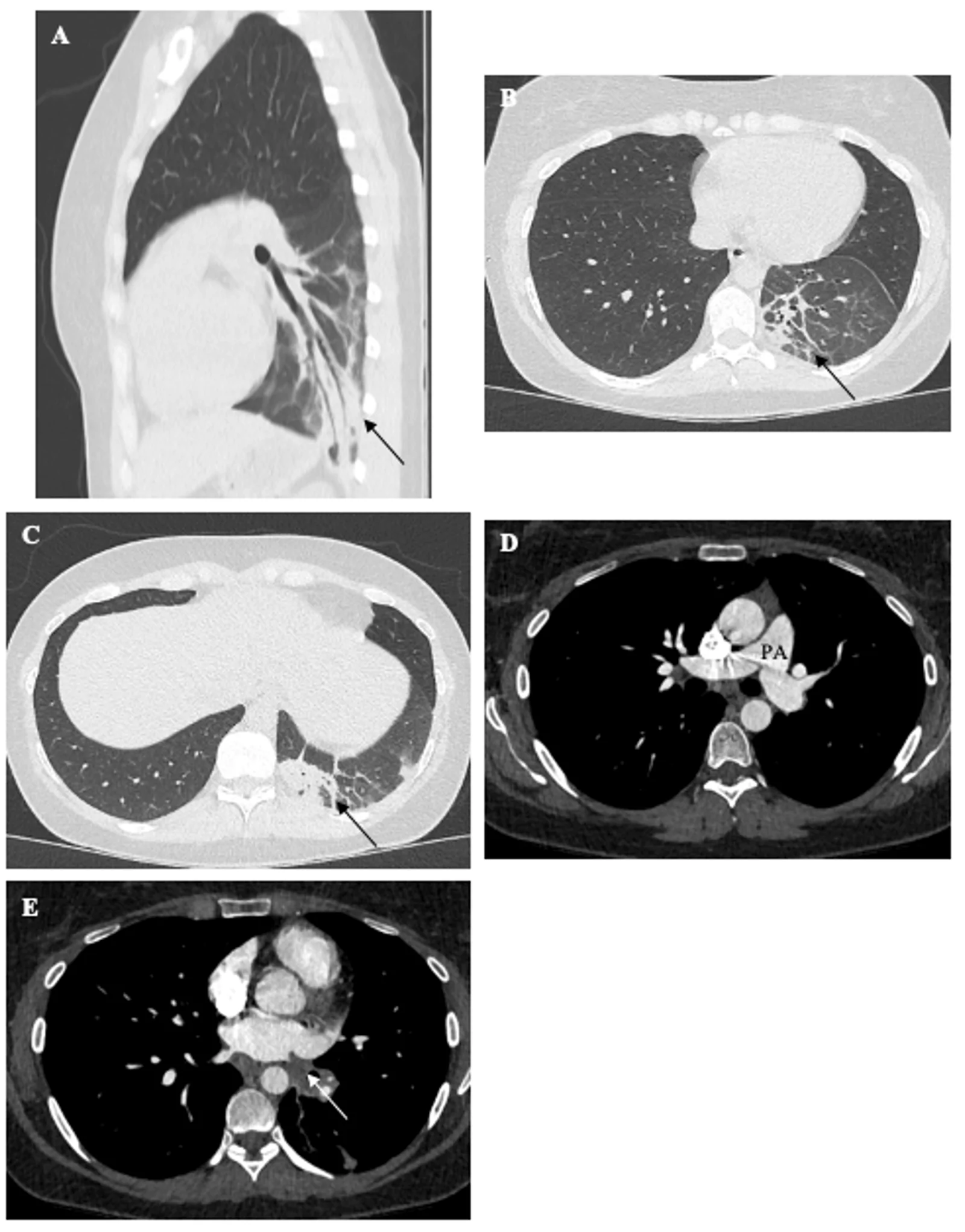
Follow-up chest computed tomography angiography (CTPA) performed 31 days post-ablation. Panels A, B, and C (axial lung window): Persistent peripheral wedge-shaped consolidation (black arrows) in the posterior segment of the left lower lobe (LLL). This finding is characteristic of a secondary venous lung infarction, demonstrating partial regression and cicatricial retraction compared to the extensive infiltrates observed at day 4. Panel D (axial mediastinal window, arterial phase): Normal and homogeneous contrast enhancement of the pulmonary arterial branches (pulmonary artery, PA), confirming the absence of concomitant arterial pulmonary embolism and definitively ruling out the initial clinical suspicion of a sequelar vascular malformation or fistula. Panel E (axial mediastinal window, arterial phase): Clear, complete intraluminal filling defect (white arrow) within the left inferior pulmonary vein (LIPV), confirming complete post-ablation pulmonary vein thrombosis.

Following this diagnosis, the patient was urgently readmitted to the cardiology department for close clinical monitoring and continuation of her therapeutic anticoagulation with enoxaparin (60 mg subcutaneously twice daily). Under this well-managed curative anticoagulation regimen, the clinical course was highly favorable, leading to the rapid and complete resolution of her chest pain and all respiratory symptoms.

## Discussion

Pulmonary vein thrombosis following AF RFCA is a rare, life-threatening, and frequently underdiagnosed complication [1-3].

The pathophysiological sequence originates with direct thermal injury to the venous endothelium during radiofrequency energy delivery. This localized trauma disrupts the antithrombotic properties of the endothelial lining and triggers an acute vascular inflammatory wall reaction [3-5]. The resulting venous outflow obstruction causes a sharp retrograde elevation in hydrostatic pressure within upstream pulmonary capillaries, inducing localized alveolar edema and hemorrhage that can evolve into secondary pulmonary venous infarction [4,7]. In chronic cases, this persistent venous congestion can spark secondary vascular remodeling, including distal venular thickening and bronchopulmonary collateral shunting [8].

As highlighted by our index case, the clinical presentation of PVT is particularly deceptive. Classic manifestations, including dyspnea, cough, chest pain, and hemoptysis, frequently mimic far more common respiratory conditions, such as infectious pneumonia, acute arterial pulmonary embolism, or bronchial neoplasia [2,3,9]. Due to this non-specific clinical presentation and a generally low index of suspicion among clinicians, both PVS and PVT are regularly misdiagnosed or attributed to alternative pulmonary pathologies. In the landmark study by Saad et al., nearly 50% of patients presenting with post-ablation pulmonary venous complications initially experienced significant diagnostic delays or erroneous therapeutic management [2]. This case underscores a paramount clinical lesson: any post-ablation respiratory symptom must systematically prompt a thorough radiological assessment of pulmonary vein patency.

In our index patient, for instance, the hemoptysis on day 4 was attributed to a simple reactive alveolar hemorrhage. This led to a diagnostic delay, as the on-call radiologist was unfamiliar with this rare complication and did not systematically evaluate the pulmonary veins. It was only on day 31, due to persistent chest pain, that the diagnosis of PVT was established. To reliably rule out PVT, appropriate imaging protocols and rigorous post-processing techniques must be implemented. Our institution's specific hemoptysis protocol consists of a non-contrast baseline scan, a pulmonary arterial phase (CTEPH protocol), and a delayed venous phase at 90 seconds. While standard arterial-phase CTPA protocols focused strictly on acute pulmonary embolism may yield sub-optimal venous enhancement, a dedicated CTEPH arterial protocol provides robust contrast opacification extending to the pulmonary arteries, aorta, and pulmonary veins, enabling direct visualization of venous patency. The complementary 90-second delayed venous phase serves to assess differential etiologies of hemoptysis. Furthermore, multiplanar reconstructions (MPR) formatted along the true anatomical long axis of each pulmonary vein ostium are critical to eliminate volume-averaging artifacts and accurately differentiate true intraluminal thrombi from flow phenomena or anatomical ostial tapering.

Furthermore, the initial clinical indication for the follow-up CT scan on day 31, to rule out a vascular malformation or sequelar fistula, reflects both the recurring diagnostic challenge and the rarity of post-ablation pulmonary venous complications compared to other pulmonary pathologies (such as acute arterial thromboembolism or pulmonary infections). It also highlights a general lack of clinical awareness regarding these rare and frequently under-recognized iatrogenic entities. Indeed, recognized iatrogenic pulmonary vascular complications following radiofrequency ablation are strictly limited to PVS, occlusion, and thrombosis [1-3].

Although iatrogenic pulmonary venous complications remain rare, a follow-up chest CT scan is strongly recommended whenever cardiorespiratory symptoms persist, or clinical deterioration occurs. Indeed, while research protocols typically schedule routine follow-up CT imaging between three and six months post-ablation [1], landmark studies such as Saad et al. and international clinical guidelines emphasize that a chest CT must be performed immediately at any time if suspicious cardiorespiratory symptoms appear or persist [2,5]. Illustrating this clinical principle, the decision to perform a chest CTPA in our patient on day 31, prompted by persistent chest pain, was entirely appropriate and clinically indicated. CTPA enables direct visualization of vascular patency, typically demonstrating a complete lack of endoluminal contrast enhancement (filling defect) within the affected pulmonary vein [8]. Timely identification of PVT is paramount. Unrecognized venous occlusion exposes the patient to irreversible parenchymal damage. Indirect parenchymal findings include localized interstitial edema (manifested as interlobular septal thickening), ground-glass opacities reflecting localized alveolar hemorrhage, and peripheral wedge-shaped consolidations (Hampton's hump) characteristic of a secondary venous lung infarction [3,4,8]. Crucially, because the thrombus resides in direct continuity with the left atrium, it poses an immediate risk of catastrophic systemic thromboembolism, such as ischemic stroke or visceral infarction [3,9].

The primary therapeutic strategy relies on rapid systemic anticoagulation (initially with heparin) followed by prolonged oral anticoagulants to arrest thrombus propagation, promote spontaneous recanalization, and prevent systemic embolization [3,9]. In the event of medical therapy failure, persistent severe PVS, or recurrent massive hemoptysis, interventional procedures such as percutaneous balloon angioplasty with bare-metal stenting [10] or salvage surgical lobectomy [7] must be considered.

## Conclusions

Post-ablation PVT remains a rare, yet grave, complication. Due to its highly nonspecific clinical presentation, systematic radiological evaluation of pulmonary vein patency, utilizing imaging protocols that ensure adequate vascular contrast enhancement, is imperative to rule out this thrombosis complication.

Radiologists should employ precisely timed acquisition phases combined with MPR aligned to the venous ostia to prevent perceptual oversight and accurately differentiate true intraluminal thrombi from artifactual flow phenomena.

Prompt initiation of therapeutic systemic anticoagulation remains the cornerstone of management. In cases of treatment failure, prompt multidisciplinary discussion is warranted to consider invasive alternatives such as percutaneous balloon angioplasty with stenting or salvage surgical lobectomy.
